# Quantitation of progenitor cell populations and growth factors after bone marrow aspirate concentration

**DOI:** 10.1186/s12967-019-1866-7

**Published:** 2019-04-08

**Authors:** Richard Schäfer, Malcolm R. DeBaun, Erika Fleck, Christopher J. Centeno, Daniela Kraft, Johannes Leibacher, Karen Bieback, Erhard Seifried, Jason L. Dragoo

**Affiliations:** 1 Institute for Transfusion Medicine and Immunohematology, German Red Cross Blood Service Baden-Württemberg – Hessen gGmbH, Frankfurt Am Main, Germany; 2 Department of Orthopedic Surgery, Stanford University School of Medicine, 450 Broadway, Redwood City, CA 94063 USA; 3 Centeno-Schultz Clinic, Broomfield, CO USA; 4 Institute of Transfusion Medicine and Immunology, Medical Faculty Mannheim, Heidelberg University, German Red Cross Blood Service Baden-Württemberg – Hessen gGmbH, Mannheim, Germany

**Keywords:** Bone marrow aspirate concentrate, Bone marrow aspiration, BMAC, Growth factors, Mesenchymal stem cell, Stem cell, Stromal cell

## Abstract

**Background:**

The number of Mesenchymal Stem/Stromal Cells (MSCs) in the human bone marrow (BM) is small compared to other cell types. BM aspirate concentration (BMAC) may be used to increase numbers of MSCs, but the composition of MSC subpopulations and growth factors after processing are unknown. The purpose of this study was to assess the enrichment of stem/progenitor cells and growth factors in BM aspirate by two different commercial concentration devices versus standard BM aspiration.

**Methods:**

120 mL of BM was aspirated from the iliac crest of 10 male donors. Each sample was processed simultaneously by either Emcyte GenesisCS^®^ (Emcyte) or Harvest SmartPReP2 BMAC (Harvest) devices and compared to untreated BM aspirate. Samples were analyzed with multicolor flow cytometry for cellular viability and expression of stem/progenitor cells markers. Stem/progenitor cell content was verified by quantification of colony forming unit-fibroblasts (CFU-F). Platelet, red blood cell and total nucleated cell (TNC) content were determined using an automated hematology analyzer. Growth factors contents were analyzed with protein quantification assays. Statistical analyses were performed by ANOVA analysis of variance followed by Tukey’s multiple comparison test or Wilcoxon matched-pairs signed rank test with p < 0.05 for significance.

**Results:**

Cell viability after processing was approximately 90% in all groups. Compared to control, both devices significantly enriched TNCs and platelets, as well as the CD45−CD73+ and CD45−CD73+CD90+ cell populations. Further, Harvest significantly concentrated CD45−CD10+, CD45−CD29+, CD45−CD90+, CD45−CD105+, CD45−CD119+ cells, and CD45dimCD90+CD271+ MSCs, whereas Emcyte significantly enriched CD45dimCD44+CD271+ MSCs. BM concentration also increased the numbers of CFU-F, platelet-derived growth factor, vascular endothelial growth factor, macrophage colony-stimulating factor, interleukin-1b, VCAM-1 and total protein. Neither system concentrated red blood cells, hematopoietic stem cells or bone morphogenetic proteins.

**Conclusion:**

This data could contribute to the development of BMAC quality control assays as both BMAC systems concentrated platelets, growth factors and non-hematopoietic stem cell subpopulations with distinct phenotypes without loss of cell viability when compared to unprocessed BM.

**Electronic supplementary material:**

The online version of this article (10.1186/s12967-019-1866-7) contains supplementary material, which is available to authorized users.

## Background

Human BM aspirate contains Mesenchymal Stem/Stromal Cells (MSCs) [1, 2], but the number of MSCs are low, estimated at 0.01–0.02% of the total cell volume [3]. Therefore, processing BM by BMAC may be clinically beneficial to increase the concentration of MSCs [4–6]. In recent years, the use of bone marrow aspirate concentrate (BMAC) has become an increasingly popular method of augmenting bone [7, 8] and cartilage regeneration [1] in orthopaedic surgery. BMAC is obtained through density gradient centrifugation of bone marrow (BM) typically aspirated from the iliac crest. It is currently approved by the United States Food and Drug Administration for clinical use as a means of acquiring progenitor cells and growth factors to promote healing in the orthopaedic patient [2].

A combination of different markers has been suggested to characterize MSCs present within BM, but especially in vitro expanded MSCs, e.g. CD45−, CD90+, CD73+, and CD271+ [5]. However, the exact phenotype of MSCs has not been agreed on yet due to the intrinsic heterogeneity of BM being comprised of different cell subsets and likely variable differentiation stages of the MSCs. Previous studies have reported considerable donor–donor variation that increases the heterogeneity of MSC phenotypes [5]. Consequently, clinical trials employing MSCs are inconsistent, hampering their broad translation into clinical practice. It is imperative to compare BMAC produced by commercially available systems to better understand its therapeutic potential and to eventually establish a correlation between the progenitor cell composition, the concentration of biologic factors and clinical outcome.

The purpose of this study is to analyze and compare progenitor cell composition and growth factors in BMAC prepared from single donors and processed concurrently by two commercially available systems versus unprocessed marrow aspiration. We hypothesized that the BMAC systems will concentrate progenitor cell populations when compared to unprocessed BM without a meaningful loss of cell viability.

## Methods

### Bone marrow aspirate collection and processing

After ethical approval and informed consent was obtained, 120 mL (60 mL per iliac crest) of bone marrow aspirate (BMA) was harvested from the anterior iliac crests of 10 male adult human donors (age range 28–35 years) using multiple puncture sites. Four mL of aspirate was withdrawn into a 60 mL syringe at each site into a syringe containing 1000 U/mL of heparin (Sigma-Aldrich, St. Louis, MO, USA). This process was repeated after the side-port Jamshidi needle was rotated 180 degrees and was withdrawn 1 cm until a total volume of 60 mL BM was obtained. This process was then repeated using the contralateral iliac crest and then consolidated into one BM sample of 120 mL per donor. Donors with conditions that could potentially compromise BM quality, including history of hematologic malignancy, current chemotherapy, BM suppressive and anti-platelet medications, previous BMA, or acute illness where excluded from this study.

Under sterile laboratory conditions, the BMA was agitated with a shaker and was alloquated on a rotating basis into the two experimental groups, which totaled 55 mL aliquots for each BMAC system, i.e. Emcyte GenesisCS^®^ (Fort Myers, FL, USA) and Harvest SmartPReP2 BMAC^®^ (Lakewood, CO, USA), and processed per the manufacturer’s instructions.

Company representatives were present ensuring proper protocols were followed. 10 mL of BMA was randomly allocated into the unprocessed control group, and served as internal control for the respective donor/experiment. Each system was adjusted to produce 7 mL of BMAC. All samples were re-suspended in Alpha MEM media (Lonza, Basel, Switzerland) and 1% Antibiotic–Antimycotic (Gibco, Grand Island, NY, USA) (7 mL BMAC in a total volume of 30 mL; 10 mL BMA in a total volume of 15 mL).

### Quantification of blood cells

Red blood cells (RBCs), platelets and total nucleated cells (TNCs) were quantified in each group using the Sysmex XT-1800*i* Automated Hematology Analyzer (Kobe, Japan).

### Flow cytometry analyses

Flow cytometry was performed to assess the impact of BMAC on important MSC populations [6–8]. Before flow cytometry analysis, BMA cells were washed with PBS and mononuclear cells were separated using Biocoll Separating Solution (Biochrom GmbH, Berlin, Germany) by centrifugation at + 4 °C, without brake for 25 min. at 400×*g*. To detach cells possibly adherent to the tube, they were incubated with 0.25% trypsin/EDTA (Gibco) for 5 min. at + 37 °C. After trypsin inactivation with phosphate buffered saline (PBS–DPBS; Gibco) + 10% fetal calf serum (PAN-Biotech GmbH, Aidenbach, Germany) they were added to the BMA samples prior to Biocoll separation. RBC lysis was performed by incubating the cells for 25 min. at + 37 °C with Red Blood Cell Lysing Buffer (Sigma, Steinheim, Germany). After an additional washing step with PBS, multicolor staining was performed at the manufacturer’s recommended concentrations of specific primary antibodies or isotype controls. Non-labeled primary antibodies were detected by secondary antibody, goat-anti mouse-PE after incubation on ice for 20 min (Table 1). Via-Probe™-7AAD solution (BD Biosciences San Diego, CA, USA) was added to determine cell viability. To identify progenitor cells and MSC subpopulations we applied combinations of markers that are commonly accepted as being either present or absent on MSCs (“mandatory markers”). In addition, as a novel approach, we tested the co-expression of markers that have been described to identify MSC subpopulations (“optional markers”) (Table 1). Flow cytometry was performed using the LSRFortessa™ cell analyzer (BD Biosciences) and data were analyzed using FACSDiva™ software (BD Biosciences). In order to detect rare stem cell subpopulations for each sample 1 × 10^6^ events were analyzed. For gating strategy see Additional file 1: Figure S1.

**Table 1:** Antibodies used for flow cytometry and rationale for marker selection

				Rationale for analysis	
Specific antibody	Fluoro-chrome	Clone	Manufacturer	Mandatory Marker presence/absence identify MSCs (include “ISCT markers”)	Optional Marker presence refine MSC subpopulations phenotypes
Anti-human CD29	PE	MAR4	BD Biosciences	Present in vitro (ISCT consensus) [9]	
Anti-human CD44	APC	DB105	Miltenyi Biotec (Bergisch-Gladbach, Germany)	Present in vitro [5]	
Anti-human CD73	APC	AD2	Biolegend (San Diego, CA, USA)	Present in vitro (ISCT consensus) and in vivo [4, 5, 28]	
Anti-human CD90	APC	5E10	BD Biosciences	Present in vitro (ISCT consensus) and in vivo [5, 6, 28]	
Anti-human CD90	PE	5E10	BD Biosciences		
Anti-human CD105	purified	266	BD Bioscience	Present in vitro (ISCT consensus) [28]	
Anti-human CD34	FITC	581	Molecular Probes Life Technologies (Carlsbad, CA, USA)	Absent in vitro (ISCT consensus) [28]; identifies HSC	
Anti-human CD45	FITC	HI30	BD Biosciences	Absent in vitro (ISCT consensus) [28]; identifies WBC and HSC	
Anti-human CD10	PE	HI10A	BD Biosciences		In vitro and in vivo [4, 5]
Anti-human CD119	PE	GIR-208	BD Biosciences		In vitro [5]
Anti-human CD271	PE	ME20.4-1.H4	Miltenyi Biotec		In vitro and in vivo [4–6]
Anti-human GD2	purified	14.G2A	BD Bioscience		In vitro and in vivo [4, 5]
Goat anti-mouse	PE	Polyclonal	BD Bioscience		
IgG1	FITC	MOPC-21	BD Biosciences		
IgG1	PE	MOPC-21	BD Biosciences		
IgG1	APC	MOPC-21	BD Biosciences		

### Colony-forming unit-fibroblasts (CFU-F) Assay

To functionally assess non-hematopoietic stem/progenitor cell content CFU-F assays were performed. BMAC and control cell suspensions were applied to Biocoll Separating Solution (Biochrom GmbH) and separated by density gradient centrifugation without break at 400×*g* for 20 min. Mononuclear cells were washed twice with PBS followed by centrifugation at 400×*g* for 5 min. The cell pellets were re-suspended in Alpha MEM (Lonza) supplemented with 10% human platelet lysate (German Red Cross Blood Service Baden-Württemberg—Hessen gGmbH, Frankfurt, Germany), 1x Gibco^®^ Antibiotic–Antimycotic (Life Technologies, NY, USA), 2 IU/mL Heparin-Natrium 5000 (Ratiopharm, Ulm, Germany) and seeded at a density of 5 × 10^5^ cells per well into 6 well plates (Thermo Fischer Scientific Nunc A/S, Roskilde, Denmark). Cells were incubated at 37 °C with 5% CO_2_. The media was changed every 3 days, and the cultures were evaluated after 10 days microscopically. CFU-F were defined as a minimum of 50 cells per CFU-F. Colonies were counted in replicates and subsequently compared as mean data for each condition and donor.

### Enzyme-linked immunosorbent assay (ELISA)

For quantification of growth factors and protein content in the cells, mononuclear cells from control and both BMAC groups, after Biocoll separation, were lysed (3 freeze/thaw cycles), and the lysate supernatants were then analyzed using the Quantibody-Array QAH-BMA-1000-2 (RayBiotech, Norcross, GA, USA) and ELISA assays (Peprotech, Hamburg, Germany) following the manufacturer’s instructions.

### Statistical analyses

In order to account for donor-to-donor variability and to achieve data comparability between flow cytometry experiments, the percentages of viable antigen(s) positive/negative cells of the BMAC groups were divided by the percentages of the corresponding controls for each experiment. The calculated ratios represent the specific fold changes for each tested marker, or marker combination, of BMAC compared to the respective internal control (single donor).

To get an estimate about the respective cell numbers, percentages of subpopulations related to the recorded viable cell counts are presented in Additional file 2: Table S1.

The statistical analyses were performed by ANOVA analysis of variance followed by Tukey’s multiple comparison test, or Wilcoxon matched-pairs signed rank test using GraphPad Prism (La Jolla, CA, USA). Differences were considered significant when p < 0.05. Presented data for blood cell counts, CFU-F and growth factors were calculated of original non-diluted samples based on analyses of diluted samples considering respective sample dilutions (both BMAC: 4.29×, controls: 1.50×) to quantify and compare actual contents.

## Results

**Fig. 1:**
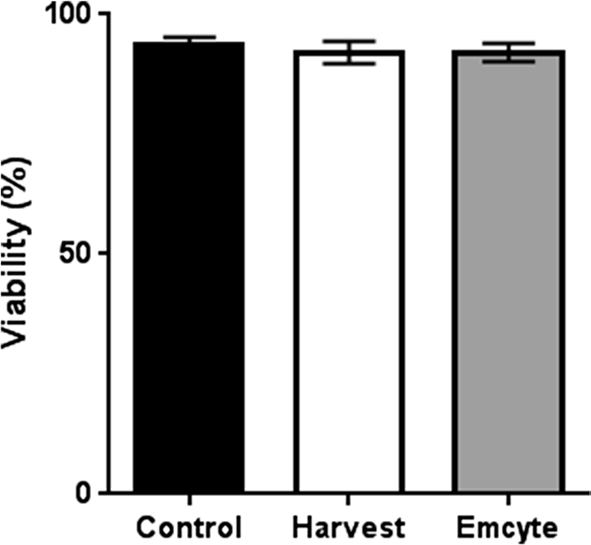
Cell viability in both BMAC groups and controls. N = 10 donors; ANOVA analysis of variance; error bars: standard error of means (SEM)

Mean cell viability after processing was similar for unprocessed controls (91.57%), Harvest (89.71%) and Emcyte (92.29%) systems (p > 0.05) (Fig. 1).

**Fig. 2:**
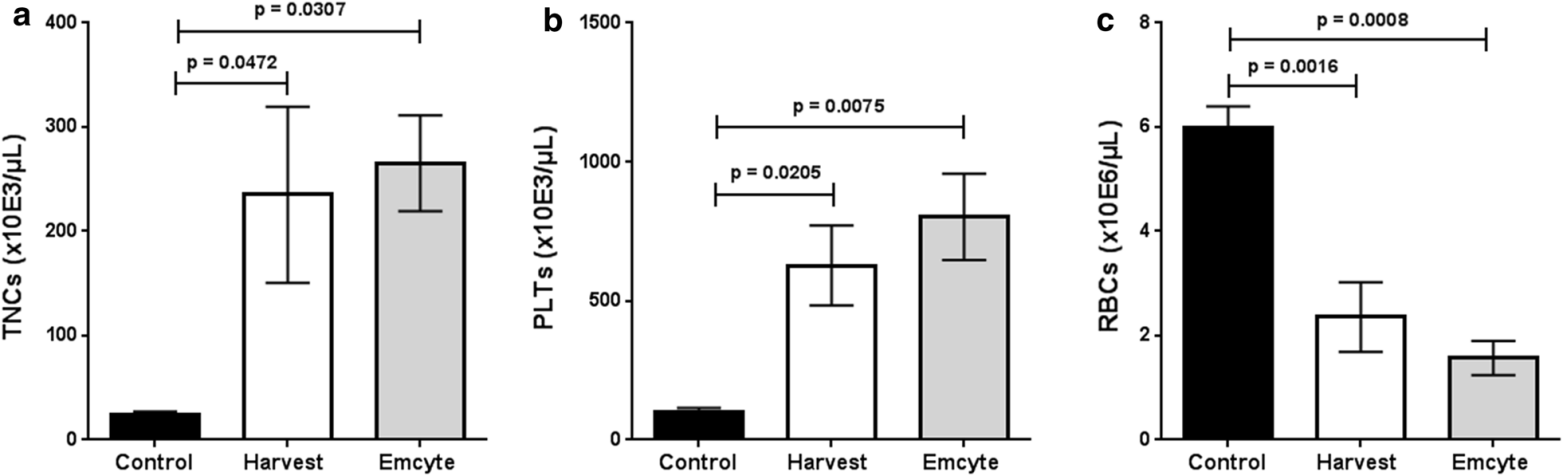
Blood cell counts in both BMAC groups and controls. N = 3 donors; ANOVA analysis of variance; error bars: SEM

Concentrations of TNCs were significantly higher in both BMAC groups compared to controls indicating an approx. tenfold concentration (Harvest: 235.11 × 10^3^/µL, p = 0.0472; Emcyte: 265.28 × 10^3^/µL, p = 0.0307; control: 24.49 × 10^3^/µL) (Fig. 2a). Both BMAC groups concentrated platelets (Harvest: 627.77 × 10^3^/µL, p = 0.0205; Emcyte: 802.23 × 10^3^/µL, p = 0.0075; control: 100 × 10^3^/µL) (Fig. 2b) but depleted red blood cells (Harvest: 2.36 × 10^6^/µL, p = 0.0016; Emcyte: 1.57 × 10^6^/µL, p = 0.0008; control: 5.99 × 10^6^/µL) (Fig. 2c).

**Fig. 3:**
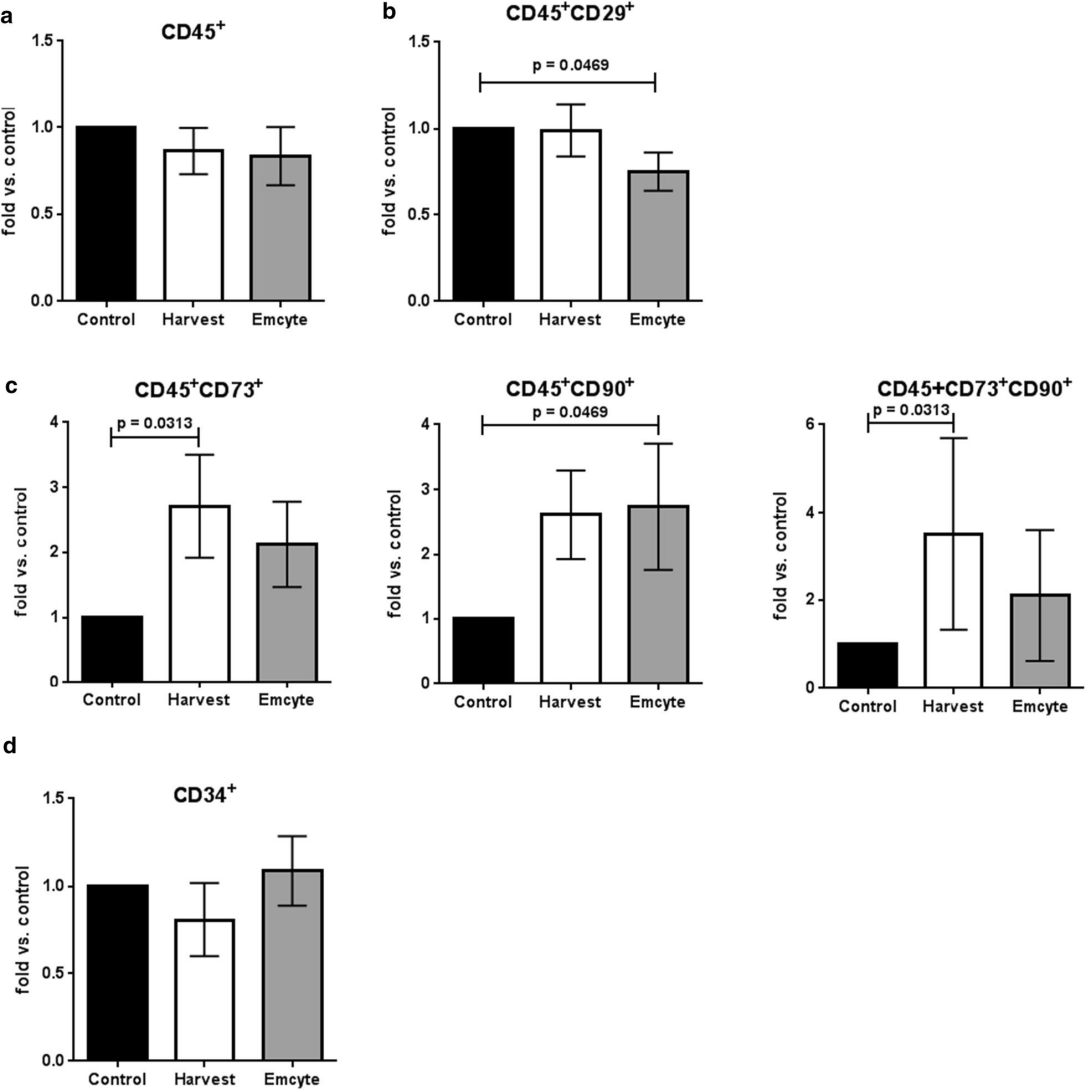
Quantification of hematopoietic (CD45+) cells in both BMAC groups versus controls. N = 7 donors (except for CD34+ [3 donors]); Wilcoxon matched-pairs signed rank test; error bars: SEM

Overall, hematopoietic cell (CD45+) content was lower in both BMAC groups compared to the unprocessed control (Harvest: 0.86 fold vs control, p = 0.5781; Emcyte: 0.83 fold vs control, p = 0.375) (Fig. 3a). Specifically, CD45+29+ cells were depleted (Harvest: 0.99 fold vs control, p = 0.9375; Emcyte: 0.75 fold vs control, p = 0.0469) (Fig. 3b). Interestingly, the content of distinct CD45+ cell populations was enriched after BM aspirate concentration, i.e. CD45+73+ (Harvest: 2.71 fold vs control, p = 0.0313; Emcyte: 2.13 vs control, p = 0.1563), CD45+90+ (Harvest: 2.61 fold vs control, p = 0.0781; Emcyte: 2.74 vs control, p = 0.0469), and CD45+73+CD90+ (Harvest: 3.51 fold vs control, p = 0.0313; Emcyte: 2.11 vs control, p = 0.0781) cells (Fig. 3c). Notably, both BMAC groups did not concentrate CD34+ hematopoietic progenitor cells (Harvest: 0.809 fold vs control, p = 0.71; Emcyte: 1.088 vs control, p = 0.93) (Fig. 3d).

In contrast, the BMAC systems enriched distinct sets of CD45− and CD45dim non-hematopoietic progenitor subpopulations. Specifically, both devices significantly enriched CD45−CD73+ (Harvest: 9.2 fold vs control, p = 0.0313; Emcyte: 11.9 fold vs control, p = 0.0469) and CD45−CD73+CD90+ (Harvest: 12.9 fold vs control, p = 0.0156; Emcyte: 7.8 vs control, p = 0.0469) cells.

**Fig. 4:**
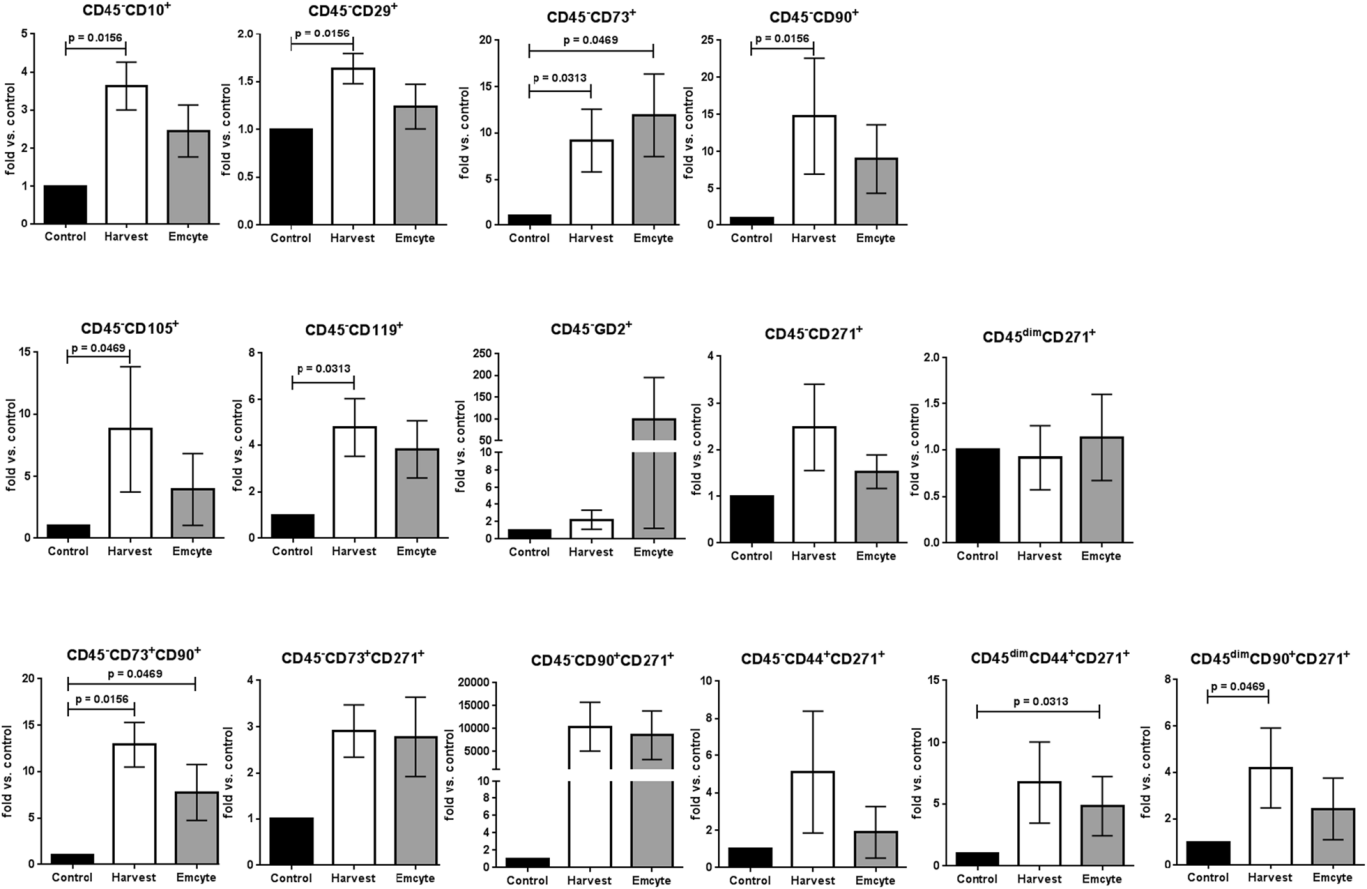
Comparison of MSC subpopulations (CD45−/dim) detected in both BMAC groups versus controls; N = 7 donors (except for CD45−CD73+ CD271 [4 donors], CD45−CD90+ CD271 [5 donors], CD45−CD44+ CD271 [5 donors], and CD45dimCD44+ CD271 [6 donors]); Wilcoxon matched-pairs signed rank test; error bars: SEM

Moreover, Harvest contained significantly more CD45−CD10+ (3.6 fold vs control, p = 0.0156), CD45−CD29+ (1.6 fold vs control, p = 0.0156), CD45−CD90+ (14.8 fold vs control, p = 0.0156), CD45−CD105+ (8.8 fold vs control, p = 0.0469), CD45−CD119+ (4.8 fold vs control, p = 0.0313) cells, and CD45dimCD90+CD271+ (4.2 fold vs control, p = 0.0469) MSCs. Emcyte significantly enriched CD45dimCD44+CD271+ (4.9 fold vs control, p = 0.0313) MSCs (Fig. 4).

In addition to stem/progenitor cell phenotype analyses by flow cytometry, the non-hematopoietic stem cell content was assessed by CFU-F assay. We observed for both BMAC a donor-depending non-hematopoietic progenitor cells enrichment between 4.4 and 41.2 fold (Table 2).

**Table 2:** Non-hematopoietic progenitor cells content in both BMAC groups and controls assessed by CFU-F assay

	Control	Harvest		Emcyte	
	CFU-F	CFU-F	Enrichment factor	CFU-F	Enrichment factor
Donor 1	127.08	407.98	4.43	1970.65	21.15
Donor 2	88.23	1758.86	19.93	3638.99	41.24
Donor 3	393.32	8194.07	22.47	9766.19	26.44

CFU-F: CFU-F total calculated per mL of original, non-diluted sample; enrichment factor: BMAC CFU-F compared to control CFU-F; data are presented as means per donor (N = 3)

**Fig. 5:**
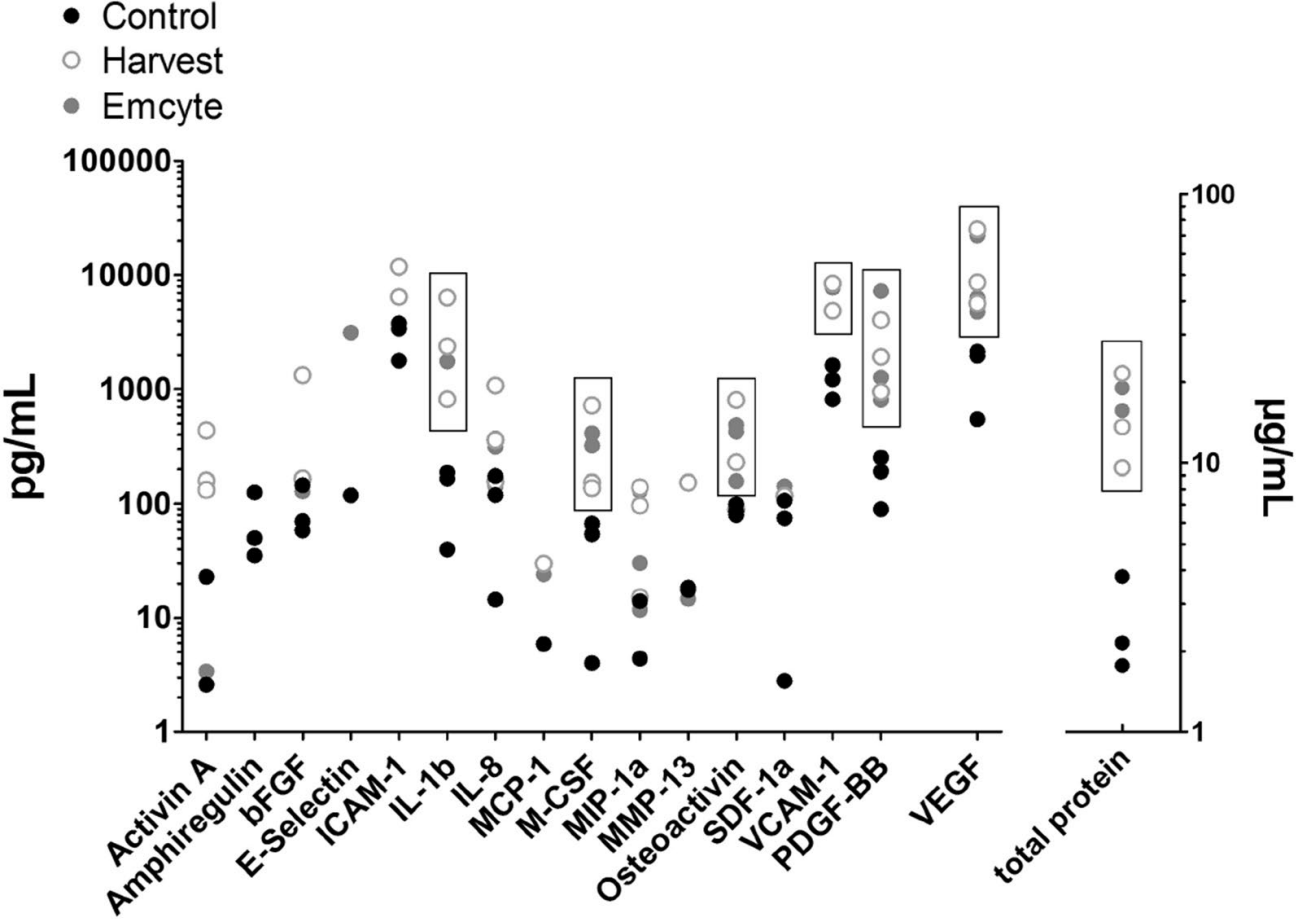
Quantification of growth factors in MNC cell lysates of both BMAC groups and controls. N = 3 donors; boxes highlight factors enriched by BMAC compared to controls. Points represent mean values for each donor

ELISA growth factor analysis of the cell lysates illustrated considerable variability between donors. Both Harvest and Emcyte concentrated platelet-derived growth factor (PDGF)-BB, vascular endothelial growth factor (VEGF), macrophage colony-stimulating factor (M-CSF), interleukin (IL)-1b, VCAM-1, osteoactivin and total protein compared to controls (Fig. 5). BMPs − 2, − 4, − 6, − 7 and − 9 were not detectable in most samples.

## Discussion

Both commercial BMAC systems significantly concentrated MSC populations, platelets and growth factors, but not hematopoietic progenitor cells, compared to untreated marrow aspirate from single donors. This is an important finding as the number of MSCs in BM is small, estimated at 0.01–0.02%, compared to other cell types. Our data corroborate other studies showing BMAC systems are effective in concentrating MSCs [9, 10].

Despite enrichment of biologic factors, the question remains as to whether higher MSC content in BMAC leads to improved clinical efficacy. Hernigou et al. [11] illustrated that concentrating BMA could increase the progenitor cell population from 612 ± 134 progenitor cells/cm^3^ before concentration to an average of 2579 ± 1121 progenitor cells/cm^3^ after processing. In their study of patients with atrophic non-unions of the tibia who received percutaneous BMAC injections, the volume of mineralization of the fracture callus at 4 months was directly related to the number of progenitor cells in the original injection, providing evidence that the efficacy of BM aspirations for fracture healing can be enhanced with BMAC [3]. Bony union was achieved in 88% of their patients at 4 months following the procedure, and they concluded that BMAC was effective as a single stage procedure to treat atrophic tibial non-unions without the risk of complications related to in vitro expansion.

Hernigou et al. [3] also illustrated that fracture healing efficiency was associated with higher CFU-F numbers, a functional assay correlated to the clone forming capacity of the cells, in BMAC. Although our study did show an increase in CFU-Fs in both BMAC groups, the previous literature suggests that CFU-F quantification alone might not be sufficient to assess BMAC’s therapeutic potential [12]. Moreover, the CFU-F assay cannot provide timely results as it requires several days ex vivo culture time.

BMAC may also promote tissue healing by delivering platelets as well as non-cellular growth factors [2, 5, 11, 13–15]. Perhaps one of the more notable findings in this study is that BMAC increases concentrations of PDGF-BB and VEGF in mononuclear cells. PDGF-BB is a potent recruiter of cells crucial to musculoskeletal tissue repair, including MSCs, osteogenic cells and tenocytes. Additionally, it up regulates angiogenesis thereby initiating a cascade of bone and soft tissue repair mechanisms in the presence of injury [16]. VEGF belongs to a subset of the PGDF family and serves as a potent initiator of angiogenesis in response to injury [17]. As we analyzed the growth factors from the lysates of the mononuclear cell fraction we hypothesize that the higher growth factors concentrations we observed in BMAC were related to the higher numbers of progenitor cells rather than platelets contents. Yet, it may be reasonable to assume that both cell types contribute synergistically as they have been shown to carry such proteins [18].

Both BMAC technologies concentrated TNCs but did not increase the overall content of hematopoietic cells (CD45+). BMAC significantly reduced (CD45+CD29+) but also enriched (CD45+73+, CD45+90+, CD45+73+CD90+) distinct subsets expressing markers that have been detected on a great variety of immune cells such as T cells, B cells, dendritic cells, and monocytes as well as on hematopoietic progenitor cells [19, 20].

Yet, to date, the effects of immune cell subset depletion or concentration by BMAC on tissue regeneration remains unclear. Additionally, the clinical relevance of specific significantly enriched CD45− (progenitor) subpopulations CD45−CD10+, CD45−CD29+, CD45−CD73+, CD45−CD90+, CD45−CD105+, CD45−CD119+, CD45−CD73+CD90+, CD45dimCD44+CD271+, and CD45dimCD90+CD271+ is unknown, especially with regards to augmenting bone and/or cartilage regeneration. CD45−CD10+, CD45−CD29+, CD45−CD119+ and CD45−CD73+CD90+ phenotypes are part of both endothelial cell and MSC marker profiles [5], but their concrete relevance to regenerative medicine is also unknown. However, the increase of CFU-F supports the MSC enrichment in both BMAC groups, and the marker combination CD45dimCD90+CD271+ specifically characterizes highly clonogenic multipotent MSCs with significant osteogenic differentiation capacity [21, 22]. This specific subpopulation was concentrated in BMAC, providing further proof of concept for its clinical use. Yet, it has to be noted that the observed increase of CD45dimCD90+CD271+ events detected by flow cytometry reflects the relationship to non-concentrated control but not absolute cell numbers. This is of particular importance as the frequency of these cells in the BM is very low, i.e. proximate to the flow cytometry detection limit. Despite this understanding, it is unclear if a greater presence of this subpopulation is relevant for clinical efficacy, as others have shown that platelets and trophic factors may also contribute to BMAC’s therapeutic effect.

Previous studies have illustrated that BMAC application resulted in significant bone and cartilage healing in both animal models and human clinical trials [2, 3, 23]. In a recent review investigating the role of BMAC in animal long bone healing, 100% of the reviewed 35 manuscripts illustrated a significant increase in radiographic bone formation in the BMAC group, while 90% showed significant early bone healing on histological analysis and 78% showed increased torsional stiffness when compared to controls [24]. In a prospective non-randomized human clinical trial, patients with large patellofemoral chondral lesions showed significantly improved clinical outcome scores at a minimum 3 year follow-up after treatment with autologous BMAC implanted into the chondral defect which was comparable to patients treated with matrix-induced autologous chondrocyte implantation [25]. In another study, patients with chondral lesions treated with BMAC plus a collagen scaffold experienced significant clinical improvement in all clinical scores compared to preoperative values, complete filling of the defect in 80% and hyaline like cartilage regeneration on histological analysis [26]. However, there is currently a lack of consensus for or against the use of a scaffold coupled with BMAC for clinical applications [27].

## Conclusion

Our data could contribute to the development of BMAC quality control assays as both commercial BMAC systems significantly concentrate MSC populations, platelets and growth factors, but not hematopoietic progenitor cells, compared to un-concentrated marrow aspirate. Clinical trials will be necessary to correlate specific MSC subpopulations and growth factors with therapeutic efficacy.

## Additional files

**Additional file 1: Figure 1.** Multicolor flow cytometry gating strategy to identify living cells (7-AAD negative), followed by identification of hematopoietic (CD45+) from non-hematopoietic (CD45−) cells with subsequent analysis (representative example). Note differences of viable CD45−CD73+CD90+ MSCs located in the upper right quadrants (asterisks) between BMAC and control.

**Additional file 2: Table S1.** Calculated percentages based on the event counts of viable cells analyzed by flow cytometry of both BMAC groups and controls.

## Abbreviations

MSC: mesenchymal stem/stromal cell(s)
BM: bone marrow
BMAC: bone marrow aspirate concentrate or concentration
BMA: bone marrow aspirate
CFU-F: colony forming unit-fibroblasts
WBC: white blood cell
RBC: red blood cell
(PDGF)-BB: platelet-derived growth factor
VEGF: vascular endothelial growth factor
M-CSF: macrophage colony-stimulating factor
VCAM-1: vascular cell adhesion protein 1
BMP: bone morphogenetic protein
ELISA: enzyme-linked immunosorbent assay
