# Supplementary figures and images for: Quantitation of progenitor cell populations and growth factors after bone marrow aspirate concentration
Source: J Transl Med. 2019 Apr 8;17:115. doi: 10.1186/s12967-019-1866-7 (PMC6454687; doi:10.1186/s12967-019-1866-7)

### Control

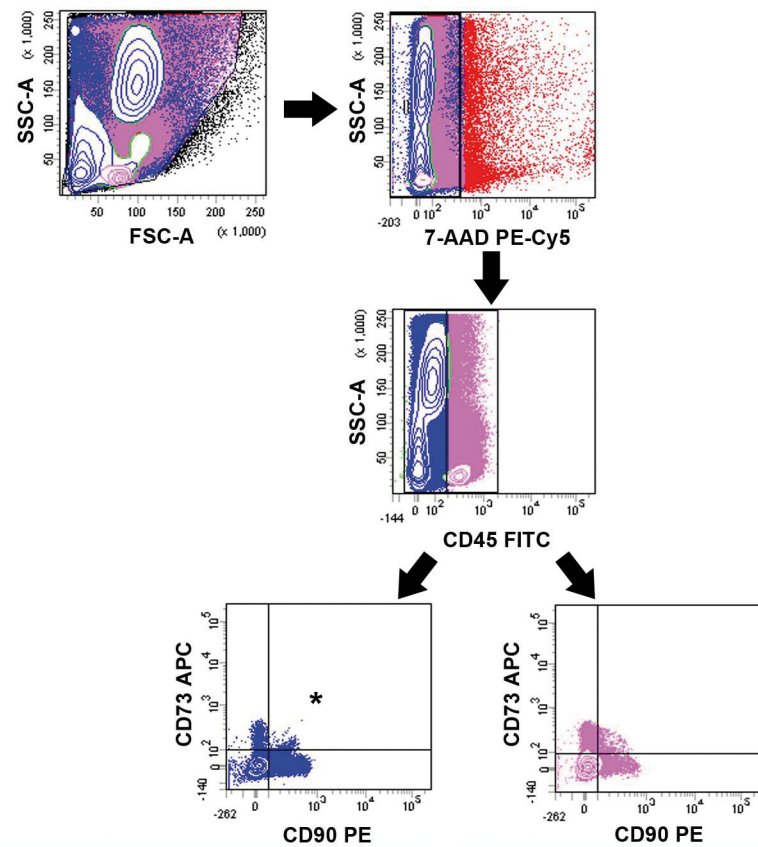

### Harvest

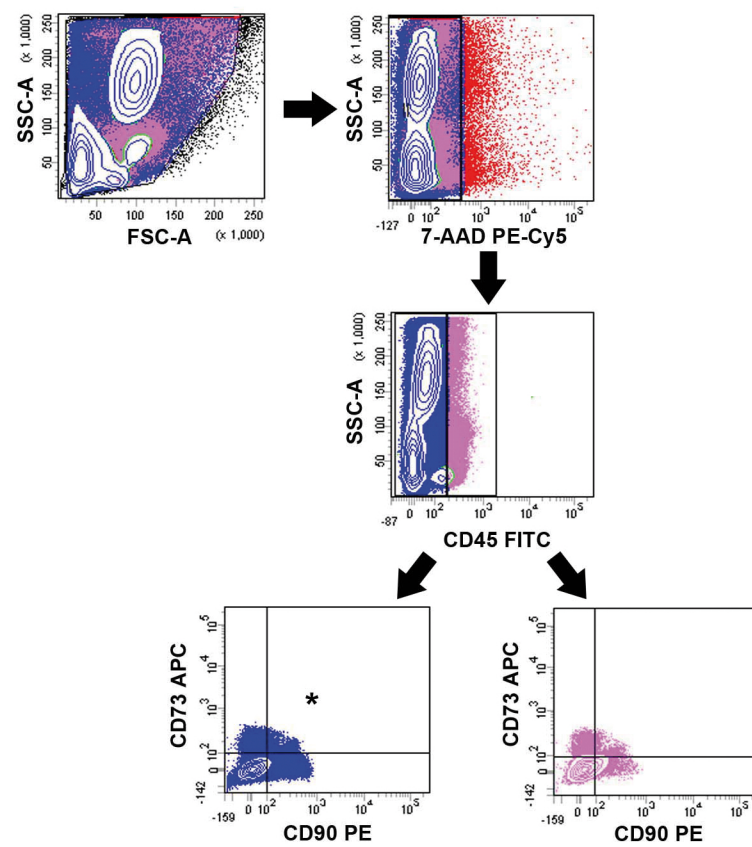

### Emcyte

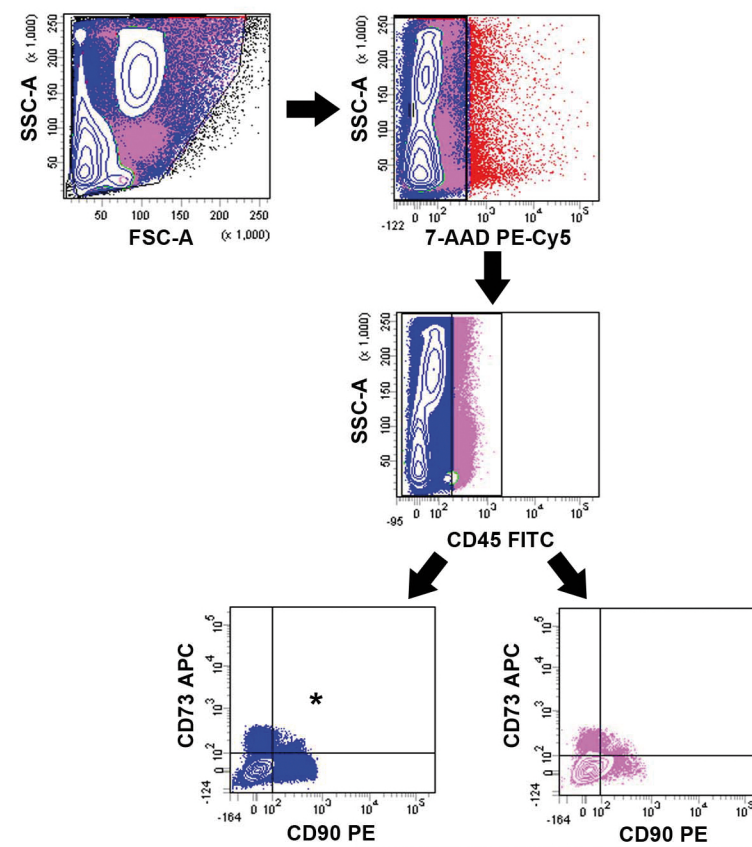

Supplement: Supplementary file 1 — Additional file 1: Figure 1. Multicolor flow cytometry gating strategy to identify living cells (7-AAD negative), followed by identification of hematopoietic (CD45+) from non-hematopoietic (CD45−) cells with subsequent analysis (representative example). Note differences of viable CD45−CD73+CD90+ MSCs located in the upper right quadrants (asterisks) between BMAC and control. [file 12967_2019_1866_MOESM1_ESM.pdf]
