# Supplementary material for: Quantitation of progenitor cell populations and growth factors after bone marrow aspirate concentration
Source: J Transl Med. 2019 Apr 8;17:115. doi: 10.1186/s12967-019-1866-7 (PMC6454687; doi:10.1186/s12967-019-1866-7)
Supplement: Supplementary file 2 — Additional file 2: Table S1. Calculated percentages based on the event counts of viable cells analyzed by flow cytometry of both BMAC groups and controls. [file 12967_2019_1866_MOESM2_ESM.docx]

**Table S1**

Calculated percentages based on the event counts of viable cells analyzed by flow cytometry of both BMAC groups and controls.

Data are presented as means; SD: standard deviation

|  |  | |  |  |
| --- | --- | --- | --- | --- |
| **Ref. to Figure 3** | **Control** | | **Harvest** | **Emcyte** |
| **CD45+ %** | **46.326** | | **39.702** | **36.877** |
| SD | 6.600 | | 4.842 | 4.711 |
| **CD29+CD45+ %** | **20.258** | | **20.850** | **16.079** |
| SD | 10.241 | | 14.074 | 12.507 |
| **CD73+CD45+ %** | **2.515** | | **6.866** | **5.153** |
| SD | 1.814 | | 5.759 | 5.400 |
| **CD90+CD45+ %** | **1.243** | | **2.856** | **2.637** |
| SD | 0.698 | | 2.485 | 2.064 |
| **CD73+CD90+CD45+ %** | **0.447** | | **1.690** | **1.223** |
| SD | 0.410 | | 2.027 | 1.484 |
| **CD34 %** | **0.883** | | **0.703** | **0.918** |
| SD | 0.167 | | 0.299 | 0.093 |
|  |  | |  |  |
| **Ref. to Figure 4** | | **Control** | **Harvest** | **Emcyte** |
| **CD10+CD45- %** | | **0.446** | **1.484** | **1.610** |
| SD | | 0.479 | 1.826 | 2.134 |
| **CD29+CD45- %** | | **12.043** | **18.681** | **18.122** |
| SD | | 6.863 | 12.729 | 14.093 |
| **CD73+CD45- %** | | **0.745** | **6.167** | **5.968** |
| SD | | 1.072 | 9.234 | 9.222 |
| **CD90+CD45- %** | | **1.029** | **4.978** | **2.569** |
| SD | | 1.092 | 5.653 | 3.161 |
| **CD105+CD45- %** | | **0.494** | **4.424** | **2.307** |
| SD | | 0.560 | 7.925 | 4.814 |
| **CD119+CD45- %** | | **2.798** | **12.300** | **11.804** |
| SD | | 3.686 | 13.321 | 14.140 |
| **GD2+CD45- %** | | **4.913** | **6.022** | **5.452** |
| SD | | 9.118 | 10.518 | 6.558 |
| **CD271+CD45- %** | | **0.034** | **0.068** | **0.063** |
| SD | | 0.029 | 0.078 | 0.091 |
| **CD45dimCD271+ %** | | **0.134** | **0.072** | **0.074** |
| SD | | 0.078 | 0.039 | 0.034 |
| **CD73+CD90+CD45- %** | | **0.185** | **2.881** | **2.664** |
| SD | | 0.176 | 3.450 | 4.209 |
| **CD73+CD271+CD45- %** | | **0.000** | **0.002** | **0.002** |
| SD | | 0.001 | 0.003 | 0.003 |
| **CD90+CD271+CD45- %** | | **0.019** | **0.003** | **0.002** |
| SD | | 0.047 | 0.003 | 0.003 |
| **CD44+CD271+CD45- %** | | **0.001** | **0.004** | **0.001** |
| SD | | 0.000 | 0.006 | 0.003 |
| **CD45dimCD44+CD271+ %** | | **0.001** | **0.003** | **0.002** |
| SD | | 0.001 | 0.004 | 0.003 |
| **CD45dimCD90+CD271+ %** | | **0.001** | **0.005** | **0.004** |
| SD | | 0.001 | 0.006 | 0.004 |
